# Training Status and Self-Reported Training Coverage of Clinical Pharmacists Across Chinese Secondary and Tertiary Hospitals: A Cross-Sectional Survey

**DOI:** 10.3390/healthcare14162622

**Published:** 2026-08-19

**Authors:** Dongting Liu, Liangjiang Chen, Xiaoyu Xi, Jing Wang

**Affiliations:** The Research Center of National Drug Policy & Ecosystem, China Pharmaceutical University, No. 639 Longmian Avenue, Jiangning District, Nanjing 211198, China; 3222041144@stu.cpu.edu.cn (D.L.); chenlj@stu.cpu.edu.cn (L.C.)

**Keywords:** clinical pharmacists, professional development, training coverage, national survey, China

## Abstract

**Highlights:**

**What are the main findings?**

- Training coverage was relatively high in core clinical pharmacy-related domains, whereas molecular biology and genomics showed comparatively limited coverage.
- Differences in training coverage were observed across training level, training type, and certification status, suggesting variations in training experiences among clinical pharmacists.

**What are the implications of the main findings?**

- The findings provide nationwide baseline evidence on the distribution and characteristics of self-reported training coverage across different knowledge and skill domains among clinical pharmacists in China.
- The findings identify knowledge and skill domains with relatively lower training coverage and support future optimization of clinical pharmacist training curricula and programs.

**Abstract:**

**Objectives**: This study aimed to characterize training coverage across different knowledge and skill domains among clinical pharmacists in China and identify variations across domains, providing nationwide evidence to inform training curriculum optimization and future development of clinical pharmacist training programs. **Methods**: A nationwide questionnaire survey was conducted using a multistage sampling method to collect data on demographic characteristics, training experiences, and self-reported training coverage across 13 knowledge and skill domains among clinical pharmacists. Descriptive statistics summarized the sample characteristics, and subgroup analyses compared differences in training coverage among clinical pharmacists with different demographic characteristics and training experiences. **Results**: A total of 704 valid questionnaires were included in the statistical analysis. Overall, pharmacists reported high training coverage in clinical pharmacy (97.30%), prescription review (94.60%) and pharmaceutical care (94.18%), whereas training coverage in molecular biology and genomics (53.27%) remained relatively limited. Subgroup analyses indicated significant differences in training coverage across multiple knowledge and skill domains by training level (national vs. provincial, *p* < 0.05), training type (general vs. specialized, *p* < 0.05), and certification status (*p* < 0.05). **Conclusions**: This study provided the first nationwide characterization of self-reported training coverage among clinical pharmacists in secondary and tertiary hospitals in China across different knowledge and skill domains. The findings provide baseline evidence on the distribution and characteristics of clinical pharmacist training coverage, identify knowledge and skill domains with relatively lower training coverage such as molecular biology and genomics, and inform future optimization of training curricula and training pathways.

## 1. Introduction

Since the concept of “pharmaceutical care” was introduced by Charles Hepler and Linda Strand in the 1990s [1], clinical pharmacists have played a crucial role worldwide by providing pharmaceutical services that improve clinical outcomes and control medication-related costs [2,3]. Their contributions to healthcare systems are increasingly recognized by the public [4].

With the continuous advancement of pharmaceutical knowledge and technology, patients’ demand for precision medicine and high-quality pharmaceutical services has grown, requiring pharmacists to continuously update their professional knowledge and skills [5]. Continuing education (CE) is essential for maintaining and updating clinical pharmacists’ professional knowledge and skills [6], and training constitutes a primary form of CE [7], enabling pharmacists to deliver higher-quality services [8,9,10]. Effective training facilitates understanding of the essence of pharmaceutical care and the evolving role of pharmacists [11,12], provides access to updated therapies and drug information [13], improves practical service capabilities [14,15,16], and strengthens pharmacists’ knowledge, attitudes [17], and behaviors to achieve better clinical outcomes [18,19,20].

Understanding the current training landscape allows for accurate assessment of training scale, structure, and regional distribution [21], informing policy adjustments and resource allocation. It also helps identify gaps in training content, align training programs with evolving clinical needs [22], and support the development of effective pharmacist training systems. Globally, diverse education and training programs have been explored [23,24,25], including integrated care pathways [26] and blended learning [27], to ensure effective pharmacist training [28,29]. Pharmacists generally demonstrate high willingness to participate in knowledge and skills training [30,31,32]. However, a 2014 study conducted by the International Pharmaceutical Federation (FIP) reported substantial disparities in clinical pharmacist training across countries [33].

In China, the clinical pharmacist training system has steadily developed [34]. Since the pilot program launched by the National Health Commission in 2005 [35] and the nationwide expansion in 2017 [36], regions have explored training models suitable for hospitals of different levels and geographic contexts. By 2022, 284 training centers had been established, training 24,443 pharmacists [37]. Despite these achievements, the current distribution and coverage of training across different knowledge and skill domains remain unclear. Variations in training content, effectiveness evaluation, faculty development, and training infrastructure across regions have resulted in insufficient pharmacist staffing, with many hospitals failing to meet national requirements [38,39]. Moreover, previous studies have suggested potential gaps between training content and evolving clinical practice needs [27,40,41].

Nationwide studies investigating the current status of clinical pharmacist training in China remain limited. Previous studies have mainly focused on macro-level aspects, including the development of clinical pharmacist training systems, establishment of training centers [42], training models [43], and policy implementation outcomes [44], or have been restricted to specific regions or training programs [45,46,47]. However, systematic evidence regarding the coverage and variation in training received by clinical pharmacists across different knowledge and skill domains remains lacking.

To address this research gap, we conducted a nationwide cross-sectional survey of clinical pharmacists across 31 provincial-level administrative regions in mainland China. Unlike previous studies focusing primarily on the development and implementation of training systems, this study focused on training content and systematically characterized self-reported training coverage across multiple knowledge and skill domains among clinical pharmacists. To improve comparability of training experiences, participants were recruited from clinical pharmacists working in secondary and tertiary hospitals in China.

This study aimed to characterize training coverage across different knowledge and skill domains among clinical pharmacists in China and identify variations across domains, providing nationwide evidence to inform training curriculum optimization and future development of clinical pharmacist training programs.

## 2. Materials and Methods

### 2.1. Study Design

#### 2.1.1. Sampling Strategy

The survey covered clinical pharmacists from 31 provincial administrative regions in mainland China (excluding Hong Kong, Macao, and Taiwan). A multistage sampling design incorporating regional stratification was adopted to achieve broad geographic and institutional coverage. Convenience sampling was applied during the recruitment of participating hospitals and clinical pharmacists.

First, all provinces, autonomous regions, and municipalities in mainland China were included. Cities within each province were categorized into high, medium, or low groups according to 2018 per capita gross domestic product (GDP), resulting in 93 city groups.

Second, because a complete sampling frame of clinical pharmacists and their affiliated medical institutions nationwide was unavailable, individual-level probability sampling could not be implemented. Therefore, within the selected survey areas, participating medical institutions were recruited using convenience sampling based on institutional willingness to participate, accessibility, and survey feasibility.

Finally, to improve hospital-level coverage, at least three eligible hospitals were included in each selected city, including at least one secondary hospital and one tertiary hospital. Within each participating hospital, eligible clinical pharmacists were recruited using convenience sampling according to their willingness to participate and the feasibility of on-site data collection. At least two clinical pharmacists were recruited from each hospital.

Inclusion criteria: full-time employed clinical pharmacists who consented to participate and were able to complete the survey (approximately 10 minutes) and provided written informed consent.

Exclusion criteria: clinical pharmacy trainees and pharmacists currently undergoing training.

#### 2.1.2. Sample Size Determination

The sample size was determined based on the predefined multistage sampling framework and the requirements of planned subgroup analyses.

According to the sampling strategy, the survey covered 93 city sampling groups across mainland China. Each sampling group was required to include at least three eligible medical institutions, with at least two clinical pharmacists recruited from each institution. Therefore, the minimum sample coverage requirement was 558 participants (93 city sampling groups × 3 hospitals × 2 clinical pharmacists).

Considering the planned subgroup analyses by training level, training type, and certification status, as well as potential heterogeneity introduced by the multistage sampling design and convenience sampling, the target sample size was increased to approximately 1100 participants to improve the stability of subgroup comparisons. Assuming an expected questionnaire completion rate of approximately 75%, approximately 1500 clinical pharmacists were invited to participate.

### 2.2. Questionnaire Development and Evaluation

The questionnaire used in this study was developed based on previous literature, clinical pharmacist training-related policy documents, and expert consultation [48,49,50,51,52,53,54,55]. It aimed to investigate clinical pharmacists’ training experiences and self-reported training coverage across different knowledge and skill domains.

The questionnaire consisted of three major sections: sociodemographic and professional characteristics, training experiences, and self-reported training coverage across knowledge and skill domains. The first two sections were developed based on previous studies and the objectives of the present study, whereas the training coverage section underwent a structured development and evaluation process involving a literature review, framework development, expert evaluation, and pilot testing.

Relevant literature and policy documents were reviewed to identify key knowledge and skill domains related to clinical pharmacist training in China. Based on these sources, the research team developed a three-level framework consisting of training domains, course categories, and specific knowledge and skill items. An initial pool of 61 items was generated from the literature review and relevant policy documents.

An expert panel consisting of seven experts with experience in clinical pharmacy education and management was invited to evaluate the questionnaire items. The panel included two hospital administrators from tertiary hospitals, two clinical pharmacy education experts, and three university faculty members specializing in clinical pharmacy. Experts evaluated questionnaire items regarding relevance, comprehensiveness, clarity, and applicability. Based on expert feedback and group discussion, items were revised, merged, or removed according to content similarity, clinical relevance, and feasibility.

Following the expert evaluation process, related items were reviewed and consolidated, resulting in 13 representative knowledge and skill domains for assessing whether clinical pharmacists had received relevant training. The detailed questionnaire items are presented in Supplementary File S1.

For each knowledge and skill domain, participants were asked: “I have attended courses or training programs in the following knowledge or skill areas.” Responses were recorded using a five-point Likert scale ranging from 1 (strongly disagree) to 5 (strongly agree), reflecting participants’ self-reported responses regarding their exposure to relevant training.

Before the formal survey, the questionnaire underwent three rounds of pretesting among eligible clinical pharmacists from tertiary hospitals in Nanjing, Jiangsu Province, to evaluate item clarity, comprehensibility, feasibility, and face validity. Subsequently, a pilot survey was conducted among 47 clinical pharmacists from 24 tertiary hospitals across six cities in Jiangsu Province to further assess the feasibility of questionnaire administration and refine the survey procedures before the nationwide investigation.

### 2.3. Data Collection

In June 2019, undergraduate students majoring in pharmacy and related disciplines were recruited as surveyors and received standardized training on the survey procedures and research ethics. The formal survey was conducted in July and August 2019 in hospitals nationwide.

After obtaining approval from participating hospitals, surveyors screened eligible participants according to the predefined inclusion criteria. Following informed consent, surveyors introduced the study purpose and procedures to participants.

Participants independently completed an anonymous electronic questionnaire through an online survey platform using mobile phones or tablets. Surveyors were responsible for questionnaire distribution, study procedure explanation, and operational assistance when needed. When participants had questions regarding the survey platform or procedures, surveyors provided operational clarification only and did not interpret, modify or influence participants’ responses.

Participation was voluntary, and participants could decline participation or discontinue the questionnaire at any time without consequences. The questionnaire did not collect directly identifiable personal information, and individual responses were kept confidential. After submission, questionnaire responses were automatically collected through the online survey platform and exported into an electronic database for subsequent data management, quality control, and statistical analysis by the research team. All electronic data were securely stored with access restricted to authorized members of the research team.

### 2.4. Data Quality Control

To ensure data quality and reliability of the analytical dataset, all returned questionnaires underwent predefined quality control procedures before statistical analysis.

Questionnaire quality control included eligibility assessment, duplicate record verification, response validity checks, and logical consistency assessments. Questionnaires that did not meet the predefined inclusion criteria were excluded. Duplicate records identified during database management were verified and excluded before statistical analysis. Response validity checks were conducted to identify and exclude invalid responses, including multiple selections in single-choice questions, out-of-range values for key variables, and violations of skip instructions. Logical consistency assessments were performed to identify unreliable responses, including implausible combinations of demographic characteristics and contradictory responses regarding training participation (e.g., reporting no training of any kind while simultaneously indicating participation in specific knowledge and skill training programs).

To further evaluate whether data quality-related exclusions could affect the characteristics of the final analytical sample, participants included in the final analysis were compared with eligible participants excluded during the quality control process. Demographic, occupational, and hospital-related characteristics were compared between the two groups, and standardized mean differences (SMDs) were calculated to assess the magnitude of between-group differences.

Following these procedures, the final dataset meeting predefined quality requirements was established for subsequent statistical analyses.

### 2.5. Statistical Analysis

#### 2.5.1. Variable Definition and Coding

Based on responses to the 13 knowledge and skill domain items in the questionnaire, self-reported training coverage was defined as whether clinical pharmacists reported having received relevant training in each specific knowledge and skill domain.

For each domain, participants responded using a five-point Likert scale. Respondents who selected “Agree” or “Strongly agree” (scores 4–5) were classified into the training covered group, indicating explicit self-reported receipt of relevant courses or training programs. Respondents who selected “Strongly disagree,” “Disagree,” or “Uncertain” (scores 1–3) were classified into the training not covered group.

This dichotomization approach was adopted because the objective of this study was to identify explicit self-reported training exposure across knowledge and skill domains rather than to assess the intensity of participants’ responses. Similar approaches have been applied in previous health-related survey studies using Likert-scale measures, in which affirmative responses were distinguished from non-affirmative responses [56,57].

Certificate categories were defined according to certificate types within the national clinical pharmacist training system in China. As the administrative authorities responsible for the national clinical pharmacist training program have changed over time, certificates issued during different periods may have different names. Therefore, certificates were grouped according to corresponding certificate types rather than issuing institutions. Accordingly, the terms “Clinical Pharmacist Training Certificate” and “Clinical Pharmacist Instructor Certificate” were used as general terms throughout the manuscript.

Definitions, categorization, and coding schemes for other covariates, including demographic characteristics, occupational variables, hospital characteristics, and training-related variables, are presented in Supplementary File S1.

#### 2.5.2. Descriptive Analysis and Subgroup Comparisons

Descriptive statistical analyses were conducted to summarize the demographic characteristics, professional characteristics, hospital-related characteristics, and training-related information of the study participants. Continuous variables were presented as means and standard deviations (mean ± SD), while categorical variables were presented as frequencies and percentages (n, %). Descriptive analyses were also performed to characterize self-reported training coverage across the 13 knowledge and skill domains.

Subgroup analyses were conducted to examine variations in self-reported training coverage according to demographic, occupational, hospital-related, and training-related characteristics, including hospital level, hospital type, gender, age, years of professional experience, professional title, educational level, training level, training type, and training certification status.

For each knowledge and skill domain, differences in training coverage (training covered/not covered) across subgroups were assessed using Pearson’s chi-square test. Fisher’s exact test was applied for comparisons involving small expected cell counts when appropriate. Cramer’s V was calculated as an effect size measure to quantify the magnitude of associations between categorical variables.

For categorical variables with statistically significant overall differences in subgroup analyses, post hoc pairwise comparisons were conducted to identify the specific groups contributing to the observed differences. Pairwise comparisons were performed using chi-square tests (or Fisher’s exact tests when appropriate), and *p* values were adjusted using the Bonferroni correction to control for type I error inflation caused by multiple comparisons. The Bonferroni-adjusted *p* values were calculated by multiplying the original *p* values by the number of pairwise comparisons performed, with adjusted *p* values greater than 1 set to 1. Statistical significance was determined based on the adjusted *p* values, with an adjusted *p* value < 0.05 considered statistically significant.

All analyses were conducted using Stata 17.0.

### 2.6. Ethical Approval

This study was approved by the Ethics Committee of China Pharmaceutical University (No. CPU2019015).

### 2.7. Data and Materials Availability

The original questionnaire and anonymized data are available from the authors upon reasonable request.

No generative artificial intelligence tools were used; only standard text editing was applied for grammar and formatting.

## 3. Results

### 3.1. Questionnaire Response

A total of 1500 clinical pharmacists were invited to participate in the survey. Among them, 1300 clinical pharmacists completed and submitted the questionnaire, resulting in a response rate of 86.67%. After eligibility assessment and predefined data quality control procedures, 704 questionnaires were included in the final statistical analysis. The detailed screening procedures and numbers of excluded questionnaires at each stage are presented in Supplementary File S2, Table S1. The final analytical sample included secondary and tertiary hospitals across 93 city/area sampling groups in mainland China, consistent with the predefined multistage sampling framework.

To further evaluate the potential impact of data quality-related exclusions on the final analytical sample, characteristics of included participants were compared with those of eligible participants excluded during the quality control process. The two groups were generally comparable in demographic characteristics, although differences were observed in some institutional and professional characteristics. Detailed results are presented in Supplementary File S2, Table S2.

### 3.2. Sociodemographic Characteristics of Clinical Pharmacists

The demographic characteristics of the surveyed clinical pharmacists are presented in Table 1. The mean age of participants was 35.69 years (*SD* = 6.62), and the mean years of professional experience was 9.52 (*SD* = 6.23). Female pharmacists accounted for 64.06% and male pharmacists for 35.94%. Most participants held junior (29.55%) or intermediate (57.24%) professional titles, while a smaller proportion held associate senior (11.51%) or senior (1.70%) titles. Educational background was primarily undergraduate (63.64%), followed by master’s degree (30.26%), and a minority held a doctoral degree (0.71%). Most pharmacists worked in general hospitals (83.38%), followed by traditional Chinese medicine/combined Chinese–Western medicine hospitals (10.94%), and only 5.68% were employed in specialized hospitals.

### 3.3. Current Status of Clinical Pharmacist Training

The training status of the surveyed clinical pharmacists is presented in Table 2. Most pharmacists had received national-level training (63.49%), while a smaller proportion had only received provincial-level training (36.51%). Regarding training type, the majority had received specialized training (54.40%), followed by general training (34.80%), and a minority had received both general and specialized training (10.80%).

Regarding certification status, participants could report more than one certificate type. Among the participants, 66.76% had obtained a Clinical Pharmacist Training Certificate, 43.18% had obtained a Clinical Pharmacist Advanced Training Certificate, and only 10.80% had obtained a Clinical Pharmacist Instructor Certificate.

Regarding self-reported training coverage across knowledge and skill domains, a high proportion of pharmacists reported receiving training in clinical pharmacy (97.30%), prescription review (94.60%), pharmaceutical care (94.18%), and pharmaceutical information retrieval and evaluation (81.82%).

### 3.4. Subgroup Analysis

The results of the subgroup analyses of self-reported training coverage across knowledge and skill domains among clinical pharmacists are presented in Table 3, Table 4, Table 5, Table 6, Table 7, Table 8, Table 9, Table 10, Table 11 and Table 12. The results of post hoc pairwise comparisons with Bonferroni-adjusted *p* values are provided in Supplementary File S2, Table S3.

#### 3.4.1. Demographic Characteristics and Self-Reported Training Coverage Across Knowledge and Skill Domains

Regarding demographic characteristics, hospital level (*p* < 0.05), hospital type (*p* < 0.01), years of professional experience (*p* < 0.01), professional title (*p* < 0.05), gender (*p* < 0.05), and age group (*p* < 0.05) were all significantly associated with differences in self-reported training coverage across knowledge and skill domains. No statistically significant differences in self-reported training coverage were observed across education levels; the results are provided in Supplementary File S2, Table S4.

Specifically, male pharmacists reported higher training coverage in pharmaceutical information retrieval and evaluation and rational use of medicines, whereas female pharmacists reported higher training coverage in pharmaceutical care.

#### 3.4.2. Training Experience and Self-Reported Training Coverage Across Knowledge and Skill Domains

Significant differences in self-reported training coverage across knowledge and skill domains were observed among pharmacists with different training experiences (*p* < 0.05):

Training Level: Compared with pharmacists who received only provincial-level training, those who received national-level training reported higher training coverage in clinical pharmacy and evidence-based medicine. Conversely, pharmacists with only provincial-level training reported higher training coverage in pharmaceutical information retrieval and evaluation, rational use of medicines, pathology and therapeutics, traditional Chinese medicine, and pharmacoepidemiology compared with those who received national-level training.

Training Type: Pharmacists who received only general training reported higher training coverage in rational use of medicines than those who received only specialized training. Pharmacists reporting experience of both general and specialized training showed broader training coverage across several knowledge and skill domains than those reporting experience of only general training or only specialized training, including pharmaceutical information retrieval and evaluation, rational use of medicines, pathology and therapeutics, traditional Chinese medicine, pharmacoepidemiology, evidence-based medicine, and molecular biology and genomics.

#### 3.4.3. Training Certificates and Self-Reported Training Coverage Across Knowledge and Skill Domains

Pharmacists holding different training certificates also showed significant differences in self-reported training coverage across knowledge and skill domains (*p* < 0.05):

Pharmacists holding a Clinical Pharmacist Training Certificate or a Clinical Pharmacist Instructor Certificate reported higher training coverage in interpersonal communication and consultation than those without the corresponding certificates.

Pharmacists holding a Clinical Pharmacist Advanced Training Certificate reported higher training coverage in pharmaceutical information retrieval and evaluation, rational use of medicines, pathology and therapeutics, traditional Chinese medicine, pharmacoepidemiology, and molecular biology and genomics than those without the certificate.

## 4. Discussion

This nationwide cross-sectional survey systematically characterized self-reported training coverage across different knowledge and skill domains among clinical pharmacists in China. Overall, clinical pharmacists from secondary and tertiary hospitals reported high training coverage in core domains, particularly clinical pharmacy, prescription review, and pharmaceutical care, whereas training coverage in emerging fields such as molecular biology and genomics was relatively lower. Significant variations in training coverage across multiple knowledge and skill domains were also observed among pharmacists with different training levels, training types, and training certificate statuses.

### 4.1. Training Coverage Across Different Knowledge and Skill Domains

In recent years, hospital pharmacy practice in China has gradually shifted from a traditional pharmaceutical supply-oriented model toward a patient-centered clinical pharmacy service model emphasizing rational medication use and pharmaceutical care. Since the implementation of the clinical pharmacist training pilot program in 2005, China has progressively established a structured clinical pharmacist training system based on training centers, standardized training programs, and clinical practice development. This study found that the highest self-reported training coverage was observed in clinical pharmacy (97.30%), prescription review (94.60%), and pharmaceutical care (94.18%), suggesting relatively broad training coverage in foundational knowledge and skill domains closely related to clinical pharmacy practice and pharmaceutical care.

From an international perspective, several domains with high training coverage among Chinese clinical pharmacist education are highly consistent with international pharmacist competency frameworks. The FIP Global Competency Framework emphasizes pharmaceutical care, professional development, and pharmacy practice competencies [58], while the American College of Clinical Pharmacy (ACCP) clinical pharmacist competency framework highlights direct patient care, pharmacotherapy knowledge, communication skills, and continuing professional development [59]. In addition, the American Society of Health-System Pharmacists (ASHP) pharmacy residency training system places considerable emphasis on patient care, medication therapy management, and clinical practice competency development [54]. Therefore, the domains with higher training coverage in this study, including clinical pharmacy, pharmaceutical care, and prescription review, are generally aligned with the core directions of international clinical pharmacist education and training.

However, despite international developments in precision medicine, molecular biology and genomics showed the lowest training coverage among the knowledge and skill domains assessed in this study. In recent years, international pharmacist education systems have increasingly emphasized pharmacogenomics (PGx) and precision medicine-related training. For example, ASHP has issued a statement regarding the role of pharmacists in the clinical implementation of pharmacogenomics [60], and PGx-related content has gradually been incorporated into Doctor of Pharmacy curricula and pharmacist training programs [61].

The relatively limited training coverage of molecular biology and genomics among Chinese clinical pharmacists may be related to several factors. First, clinical pharmacist training in China has traditionally placed greater emphasis on rational medication use, clinical pharmacy practice, and pharmaceutical care, whereas pharmacogenomics and precision medicine represent rapidly developing interdisciplinary fields, with related training systems still evolving. Second, this domain involves multidisciplinary knowledge, including genetics, molecular biology, bioinformatics, and interpretation of genetic testing results, which may require additional expertise, curriculum development, and clinical implementation support [62]. In addition, insufficient PGx training and limited confidence in clinical application have also been reported among pharmacists internationally, particularly in regions with limited educational and healthcare resources [63,64].

Future development of clinical pharmacist training programs in China should further incorporate precision medicine-related content, including molecular biology, pharmacogenomics, and personalized medication therapy, into structured training curricula. Moreover, interdisciplinary collaboration and practice-oriented education involving clinical pharmacy, genetics, molecular diagnostics, and bioinformatics should be strengthened to enhance clinical pharmacists’ ability to participate in precision medication decision-making.

### 4.2. Training Coverage Across Knowledge and Skill Domains Among Clinical Pharmacists with Different Training Levels

By the end of 2023, 304 clinical pharmacist training centers had been established nationwide [65], mainly including national-level and provincial-level training centers. These two types of training centers differ in accreditation authorities, resource conditions, training objectives, and service orientations. However, systematic comparisons of training coverage across knowledge and skill domains among clinical pharmacists receiving training at different levels remain limited.

This study found significant differences in training coverage across several knowledge and skill domains among clinical pharmacists with different training levels. Higher self-reported training coverage in clinical pharmacy and evidence-based medicine was observed among pharmacists receiving national-level training, whereas higher self-reported coverage in pharmaceutical information retrieval and evaluation, rational use of medicines, pathology and therapeutics, traditional Chinese medicine, and pharmacoepidemiology was observed among those receiving only provincial-level training. These differences may partly reflect variations in training resources, educational orientations, and curriculum priorities across different levels of training centers.

Differences in training resources and educational conditions may partly explain these findings. National-level training centers may have greater access to faculty resources and educational platforms, and may place greater emphasis on areas requiring advanced training support, such as clinical pharmacy and evidence-based medicine [66], which may contribute to higher reported training coverage in related domains. In contrast, provincial-level training centers may be more closely connected with local healthcare contexts and practical service requirements, which may contribute to relatively higher training coverage in certain knowledge and skill domains related to routine clinical practice [67].

These findings should not be interpreted as indicating that one training level is superior to another; rather, they may reflect differences in training objectives, resources, and service needs. Further coordination between training levels may help optimize curriculum design and improve balance in training coverage across knowledge and skill domains.

### 4.3. Training Coverage Across Knowledge and Skill Domains Among Clinical Pharmacists with Different Training Types

By the end of 2023, China had implemented a clinical pharmacist training system comprising 20 specialized programs and one general program [65], with national guidelines for general and specialized training [44,67]. However, systematic comparisons of training coverage across different knowledge and skill domains among clinical pharmacists receiving different types of training remain limited.

This study found that pharmacists who received only general training reported higher self-reported training coverage in rational use of medicines than those who received only specialized training. This difference may reflect variations in training objectives, curriculum orientations, and knowledge and skill development priorities between general and specialized training. General training primarily aims to establish a broad foundation in clinical pharmacy knowledge and general pharmaceutical care skills, whereas specialized training focuses on in-depth development within specific disease areas and advanced clinical practice skills [68]. Therefore, general training may provide broader exposure to principles of rational drug use and basic pharmacotherapy, potentially contributing to higher self-reported training coverage in this domain [69].

Furthermore, pharmacists with experience of both general and specialized training reported broader training coverage across multiple knowledge and skill domains. This may reflect the complementary nature of these two training pathways, with general training providing foundational knowledge and specialized training extending disease-specific clinical practice exposure. Strengthening coordination between different training stages and optimizing curriculum design may help promote a more systematic and continuous clinical pharmacist training system.

### 4.4. Training Coverage Across Knowledge and Skill Domains Among Clinical Pharmacists with Different Training Certificates

This study found that clinical pharmacists holding specific training certificates reported higher self-reported training coverage in several knowledge and skill domains compared with those without corresponding certificates. These findings suggest that training certificate status may be associated with variations in self-reported training coverage across knowledge and skill domains. However, the association between training certificate status and training coverage across knowledge and skill domains remains insufficiently investigated.

Clinical pharmacists holding the Clinical Pharmacist Training Certificate or the Clinical Pharmacist Instructor Training Certificate reported higher training coverage in the domain of interpersonal communication and consultation skills than those without these certificates. This difference may reflect the emphasis placed on communication skills in the objectives of these training programs. Clinical pharmacists require not only pharmacotherapy-related knowledge but also effective communication and collaboration skills when interacting with patients, physicians, and other healthcare professionals. Therefore, interpersonal communication and consultation skills have been recognized as important components of core competencies for clinical pharmacists in both national and international consensus statements [70,71,72,73,74]. Previous studies have reported that training programs associated with these certificates include structured components related to communication skills [75,76,77], which may partly explain the observed differences in training coverage [78,79,80].

Notably, clinical pharmacists holding the Clinical Pharmacist Advanced Training Certificate reported higher training coverage across multiple knowledge and skill domains. This finding may reflect the characteristics of advanced training programs, which may provide more practice-oriented learning opportunities and broader exposure to specialized knowledge domains. The inclusion of traditional Chinese medicine-related content may also support the integration of traditional Chinese and Western medicine knowledge in pharmaceutical practice [81].

These findings highlight the need to further clarify the roles of different training certificates and strengthen coordination among training programs to optimize curriculum design and resource allocation within the clinical pharmacist training system.

### 4.5. Policy Recommendations

Based on the findings of this study, future development of the clinical pharmacist training system in China should focus on three aspects: training content, training models, and evaluation mechanisms.

First, training content should be dynamically updated according to evolving clinical practice needs. With the development of precision medicine and pharmacogenomics, emerging interdisciplinary knowledge should be gradually incorporated into training curricula. In particular, molecular biology and genomics-related content, which showed relatively lower training coverage in this study, should receive greater attention in future training programs.

Second, a more coordinated and progressive training model should be established. The roles of different levels of training centers should be further clarified, resource sharing should be promoted, and the coordination between general and specialized training should be strengthened to support the continuous professional development of clinical pharmacists.

Finally, training evaluation mechanisms should be further improved. Future evaluations should move beyond training completion and incorporate objective assessment approaches, such as knowledge assessments, case-based assessments, and structured clinical evaluations. Continuous quality improvement mechanisms should also be established to optimize training content and educational approaches.

### 4.6. Limitations

This study has several limitations. First, convenience sampling was used during the multistage survey process, which may have introduced selection bias and limited the generalizability of the findings. Although this study covered all 31 provincial-level administrative regions in mainland China and included clinical pharmacists from both secondary and tertiary hospitals, the possibility of selection bias cannot be completely excluded. The demographic characteristics and educational background of the study sample were broadly comparable with those reported in previous nationwide studies of clinical pharmacists in China [82], supporting the broad comparability of the study sample. Therefore, the findings should be interpreted primarily as a nationwide characterization of self-reported training coverage among clinical pharmacists in secondary and tertiary hospitals. Second, the data in this study were based on self-reported responses and may have been subject to recall bias and social desirability bias, which could have influenced the accuracy of reported training coverage. Therefore, the findings primarily reflect self-reported training coverage among clinical pharmacists and may not fully represent objectively documented training experiences or professional performance. Future studies could incorporate training records and objective assessments of knowledge or skills to further evaluate training exposure and its relationship with clinical practice. Third, due to the cross-sectional design, this study could only describe associations between individual characteristics, training experiences, and self-reported training coverage across knowledge and skill domains. Therefore, causal relationships between training level, training type, certificate status, and variations in training coverage could not be established. In addition, this study could not assess changes in training coverage over time. Future longitudinal studies are needed to explore how training coverage evolves and how different training experiences are associated with these changes. Finally, the data were collected from July to August 2019, before the coronavirus disease 2019 (COVID-19) pandemic, and therefore mainly reflect the pre-pandemic training coverage of clinical pharmacists across different knowledge and skill domains in China. Since training policies, program structures, and delivery modes may have evolved in recent years, the findings may not fully represent the current training landscape. Nevertheless, this nationwide survey provides valuable baseline information on clinical pharmacist training coverage and may serve as a reference for future evaluations of changes in training systems.

## 5. Conclusions

This study provided the first nationwide systematic assessment of self-reported training coverage across different knowledge and skill domains among clinical pharmacists in secondary and tertiary hospitals in China. By characterizing variations in training coverage across domains and training experiences, this study provides baseline evidence for understanding the distribution and characteristics of clinical pharmacist training content in China and may inform curriculum optimization, training program development, and future evaluation strategies.

Future studies should build upon this nationwide baseline and conduct longitudinal investigations to explore changes in clinical pharmacist training content over time. In addition, incorporating objective assessments and training records may help further clarify the associations between knowledge and skill development, training experiences and clinical practice.

## Figures and Tables

**Table 1:** Demographic characteristics of surveyed clinical pharmacists (*N* = 704).

Variable	Mean	SD	*N*	%
**Age**	35.69	6.62		
**Years of professional experience**	9.52	6.23		
**Hospital level**				
Secondary			375	53.27%
Tertiary			329	46.73%
**Hospital type**				
General hospital			587	83.38%
Specialty hospital			40	5.68%
Traditional Chinese medicine/integrated Chinese–Western medicine hospital			77	10.94%
**Gender**				
Male			253	35.94%
Female			451	64.06%
**Professional title**				
Junior			208	29.55%
Intermediate			403	57.24%
Associate senior			81	11.51%
Senior			12	1.70%
**Highest educational qualification**				
Below bachelor’s degree			38	5.40%
Bachelor’s degree			448	63.64%
Master’s degree			213	30.26%
Doctoral degree			5	0.71%

**Table 2:** Training experience of clinical pharmacists and self-reported training coverage across knowledge and skill domains (*N* = 704).

Variable	Mean	SD	*N*	%
**Training Level**				
National-level training			447	63.49%
Provincial-level training			257	36.51%
**Training Type**				
General training only			245	34.80%
Specialist training only			383	54.40%
Both general and specialist training			76	10.80%
**Training Certificates**				
Clinical Pharmacist Training Certificate			470	66.76%
Clinical Pharmacist Advanced Training Certificate			304	43.18%
Clinical Pharmacist Instructor Certificate			76	10.80%
**S** **elf-reported training coverage across knowledge and skill domain** **s**				
Prescription review			666	94.60%
Drug information retrieval and evaluation			576	81.82%
Rational drug use			410	58.24%
Clinical pharmacy			685	97.30%
Pharmaceutical care			663	94.18%
Pathology and therapeutics			517	73.44%
Computer and internet skills			446	63.35%
Interpersonal communication			470	66.76%
Traditional Chinese medicine			426	60.51%
Pharmacoeconomics			424	60.23%
Pharmacoepidemiology			473	67.19%
Evidence-based medicine			474	67.33%
Molecular biology and genomics			375	53.27%
**Number of knowledge and skill domains with reported training coverage**	9.38	3.14		

**Table 3:** Self-reported training coverage of knowledge and skill domains by hospital level.

	Hospital Level		*p* Value	Cramer’s V
	Secondary	Tertiary		
Prescription review	352 (93.87%)	314 (95.44%)	0.356	0.035
Drug information retrieval and evaluation	302 (80.53%)	274 (83.28%)	0.345	0.036
Rational drug use	205 (54.67%) *	205 (62.31%) *	0.040	0.077
Clinical pharmacy	367 (97.87%)	318 (96.66%)	0.323	0.037
Pharmaceutical care	359 (95.73%)	304 (92.40%)	0.060	0.071
Pathology and therapeutics	266 (70.93%)	251 (76.29%)	0.108	0.061
Computer and internet skills	234 (62.40%)	212 (64.44%)	0.576	0.021
Interpersonal communication	250 (66.67%)	220 (66.87%)	0.955	0.002
Traditional Chinese medicine	218 (58.13%)	208 (63.22%)	0.168	0.052
Pharmacoeconomics	240 (64.00%) *	184 (55.93%) *	0.029	0.082
Pharmacoepidemiology	243 (64.80%)	230 (69.91%)	0.150	0.054
Evidence-based medicine	264 (70.40%)	210 (63.83%)	0.064	0.070
Molecular biology and genomics	199 (53.07%)	176 (53.50%)	0.909	0.004

Note: * *p* < 0.05; (chi-square test). The table presents the number and percentage of participants with reported training coverage in each knowledge and skill domain.

**Table 4:** Self-reported training coverage of knowledge and skill domains by hospital type.

	Hospital Type			*p* Value	Cramer’s V
	General Hospitals	Specialist Hospitals	Traditional Chinese Medicine /Integrated Chinese–Western Medicine Hospitals		
Prescription review	554 (94.38%)	40 (100%)	72 (93.51%)	0.284	0.060
Drug information retrieval and evaluation	480 (81.77%)	36 (90.00%)	60 (77.92%)	0.274	0.061
Rational drug use	340 (57.92%)	25 (62.50%)	45 (58.44%)	0.850	0.022
Clinical pharmacy	574 (97.79%)	38 (95.00%)	73 (94.81%)	0.206	0.067
Pharmaceutical care	554 (94.38%)	38 (95.00%)	71 (92.21%)	0.727	0.030
Pathology and therapeutics	431 (73.42%)	31 (77.50%)	55 (71.43%)	0.780	0.026
Computer and internet skills	364 (62.01%)	29 (72.50%)	53 (68.83%)	0.235	0.064
Interpersonal communication	394 (67.12%)	29 (72.50%)	47 (61.04%)	0.414	0.050
Traditional Chinese medicine	338 (57.58%) **	27 (67.50%)	61 (79.22%) **	0.001	0.142
Pharmacoeconomics	359 (61.16%)	25 (62.50%)	40 (51.95%)	0.286	0.060
Pharmacoepidemiology	396 (67.46%)	28 (70.00%)	49 (63.64%)	0.739	0.029
Evidence-based medicine	392 (66.78%)	29 (72.50%)	53 (68.83%)	0.724	0.030
Molecular biology and genomics	306 (52.13%)	24 (60.00%)	45 (58.44%)	0.394	0.051

Note: ** *p* < 0.01 (chi-square test with post hoc multiple comparisons). The table presents the number and percentage of participants with reported training coverage in each knowledge and skill domain.

**Table 5:** Self-reported training coverage of knowledge and skill domains by gender.

	Gender		*p* Value	Cramer’s V
	Male	Female		
Prescription review	241 (95.26%)	425 (94.24%)	0.565	0.022
Drug information retrieval and evaluation	222 (87.75%) **	354 (78.49%) **	0.002	0.115
Rational drug use	163 (64.43%) *	247 (54.77%) *	0.013	0.094
Clinical pharmacy	245 (96.84%)	440 (97.56%)	0.570	0.021
Pharmaceutical care	232 (91.70%) *	431 (95.57%) *	0.036	0.079
Pathology and therapeutics	192 (75.89%)	325 (72.06%)	0.270	0.042
Computer and internet skills	153 (60.47%)	293 (64.97%)	0.235	0.045
Interpersonal communication	159 (62.85%)	311 (68.96%)	0.099	0.062
Traditional Chinese medicine	157 (62.06%)	269 (59.65%)	0.530	0.024
Pharmacoeconomics	154 (60.87%)	270 (59.87%)	0.794	0.010
Pharmacoepidemiology	172 (67.98%)	301 (66.74%)	0.736	0.013
Evidence-based medicine	178 (70.36%)	296 (65.63%)	0.200	0.048
Molecular biology and genomics	139 (54.94%)	236 (52.33%)	0.505	0.025

Note: * *p* < 0.05; ** *p* < 0.01 (chi-square test). The table presents the number and percentage of participants with reported training coverage in each knowledge and skill domain.

**Table 6:** Self-reported training coverage of knowledge and skill domains by age.

	Age				*p* Value	Cramer’s V
	18–29	30–39	40–49	≥50		
Prescription review	59 (92.19%)	443 (94.66%)	133 (94.33%)	31 (100%)	0.471	0.060
Drug information retrieval and evaluation	54 (84.38%)	379 (80.98%)	114 (80.85%)	29 (93.55%)	0.326	0.070
Rational drug use	42 (65.63%)	247 (52.78%) **	95 (67.38%) *	26 (83.87%) **	0.000	0.170
Clinical pharmacy	62 (96.88%)	458 (97.86%)	135 (95.74%)	30 (96.77%)	0.585	0.053
Pharmaceutical care	60 (93.75%)	448 (95.73%) *	126 (89.36%) *	29 (93.55%)	0.045	0.107
Pathology and therapeutics	54 (84.38%)	327 (69.87%)	112 (79.43%)	24 (77.42%)	0.020	0.118
Computer and internet skills	47 (73.44%)	287 (61.32%)	93 (65.96%)	19 (61.29%)	0.251	0.076
Interpersonal communication	45 (70.31%)	312 (66.67%)	92 (65.25%)	21 (67.74%)	0.913	0.027
Traditional Chinese medicine	42 (65.63%)	270 (57.69%)	92 (65.25%)	22 (70.97%)	0.172	0.084
Pharmacoeconomics	41 (64.06%)	278 (59.40%)	85 (60.28%)	20 (64.52%)	0.858	0.033
Pharmacoepidemiology	50 (78.13%)	298 (63.68%)	102 (72.34%)	23 (74.19%)	0.037	0.110
Evidence-based medicine	41 (64.06%)	318 (67.95%)	94 (66.67%)	21 (67.74%)	0.936	0.025
Molecular biology and genomics	44 (68.75%) *	233 (49.79%) *	82 (58.16%)	16 (51.61%)	0.020	0.118

Note: * *p* < 0.05; ** *p* < 0.01 (chi-square test with post hoc multiple comparisons). The table presents the number and percentage of participants with reported training coverage in each knowledge and skill domain.

**Table 7:** Self-reported training coverage of knowledge and skill domains by years of professional experience.

	Years of Professional Experience			*p* Value	Cramer’s V
	0–10	10–20	≥20		
Prescription review	389 (94.19%)	201 (95.26%)	76 (95.00%)	0.843	0.022
Drug information retrieval and evaluation	342 (82.81%)	165 (78.20%)	69 (86.25%)	0.203	0.067
Rational drug use	222 (53.75%) *	129 (61.14%) *	59 (73.75%)	0.002	0.131
Clinical pharmacy	403 (97.58%)	207 (98.10%)	75 (93.75%)	0.106	0.080
Pharmaceutical care	392 (94.92%)	199 (94.31%)	72 (90.00%)	0.227	0.065
Pathology and therapeutics	298 (72.15%)	155 (73.46%)	64 (80.00%)	0.347	0.055
Computer and internet skills	266 (64.41%)	124 (58.77%)	56 (70.00%)	0.163	0.072
Interpersonal communication	277 (67.07%)	136 (64.45%)	57 (71.25%)	0.535	0.042
Traditional Chinese medicine	250 (60.53%)	120 (56.87%)	56 (70.00%)	0.123	0.077
Pharmacoeconomics	252 (61.02%)	122 (57.82%)	50 (62.50%)	0.674	0.034
Pharmacoepidemiology	275 (66.59%)	142 (67.30%)	56 (70.00%)	0.837	0.023
Evidence-based medicine	280 (67.80%)	141 (66.82%)	53 (66.25%)	0.948	0.012
Molecular biology and genomics	219 (53.03%)	111 (52.61%)	45 (56.25%)	0.847	0.022

Note: * *p* < 0.05; (chi-square test with post hoc multiple comparisons). The table presents the number and percentage of participants with reported training coverage in each knowledge and skill domain.

**Table 8:** Self-reported training coverage of knowledge and skill domains by professional title.

	Professional Title				*p* Value	Cramer’s V
	Junior	Intermediate	Associate Senior	Senior		
Prescription review	197 (94.71%)	380 (94.29%)	78 (96.30%)	11 (91.67%)	0.864	0.032
Drug information retrieval and evaluation	180 (86.54%)	317 (78.66%)	68 (83.95%)	11 (91.67%)	0.077	0.099
Rational drug use	127 (61.06%)	222 (55.09%)	52 (64.20%)	9 (75.00%)	0.180	0.083
Clinical pharmacy	204 (98.08%)	391 (97.02%)	79 (97.53%)	11 (91.67%)	0.559	0.054
Pharmaceutical care	197 (94.71%)	380 (94.29%)	76 (93.83%)	10 (83.33%)	0.439	0.062
Pathology and therapeutics	157 (75.48%)	285 (70.72%)	67 (82.72%)	8 (66.67%)	0.120	0.091
Computer and internet skills	142 (68.27%)	242 (60.05%)	55 (67.90%)	7 (58.33%)	0.178	0.084
Interpersonal communication	137 (65.87%)	266 (66.00%)	59 (72.84%)	8 (66.67%)	0.676	0.047
Traditional Chinese medicine	132 (63.46%)	238 (59.06%)	48 (59.26%)	8 (66.67%)	0.715	0.044
Pharmacoeconomics	127 (61.06%)	231 (57.32%)	58 (71.60%)	8 (66.67%)	0.108	0.093
Pharmacoepidemiology	153 (73.56%) *	252 (62.53%) *	59 (72.84%)	9 (75.00%)	0.026	0.115
Evidence-based medicine	139 (66.83%)	268 (66.50%)	59 (72.84%)	8 (66.67%)	0.736	0.043
Molecular biology and genomics	115 (55.29%)	211 (52.36%)	43 (53.09%)	6 (50.00%)	0.913	0.027

Note: * *p* < 0.05; (chi-square test with post hoc multiple comparisons). The table presents the number and percentage of participants with reported training coverage in each knowledge and skill domain.

**Table 9:** Self-reported training coverage of knowledge and skill domains by training level.

	Training Level		*p* Value	Cramer’s V
	National-Level Training	Provincial-Level Training		
Prescription review	420 (93.96%)	246 (95.72%)	0.320	0.038
Drug information retrieval and evaluation	356 (79.64%) *	220 (85.60%) *	0.048	0.074
Rational drug use	234 (52.35%) **	176 (68.48%) **	0.000	0.158
Clinical pharmacy	440 (98.43%) *	245 (95.33%) *	0.014	0.092
Pharmaceutical care	426 (95.30%)	237 (92.22%)	0.093	0.063
Pathology and therapeutics	308 (68.90%) **	209 (81.32%) **	0.000	0.135
Computer and internet skills	274 (61.30%)	172 (66.93%)	0.136	0.056
Interpersonal communication	299 (66.89%)	171 (66.54%)	0.924	0.004
Traditional Chinese medicine	253 (56.60%) **	173 (67.32%) **	0.005	0.105
Pharmacoeconomics	281 (62.86%)	143 (55.64%)	0.059	0.071
Pharmacoepidemiology	286 (63.98%) *	187 (72.76%) *	0.017	0.090
Evidence-based medicine	317 (70.92%) **	157 (61.09%) **	0.007	0.101
Molecular biology and genomics	238 (53.24%)	137 (53.31%)	0.987	0.001

Note: * *p* < 0.05; ** *p* < 0.01 (chi-square test). The table presents the number and percentage of participants with reported training coverage in each knowledge and skill domain.

**Table 10:** Self-reported training coverage of knowledge and skill domains by training type.

	Training Type			*p* Value	Cramer’s V
	General Training Only	Specialist Training Only	Both General and Specialist Training		
Prescription review	229 (93.47%)	363 (94.78%)	74 (97.37%)	0.411	0.050
Drug information retrieval and evaluation	208 (84.90%)	299 (78.07%) *	69 (90.79%) *	0.010	0.115
Rational drug use	152 (62.04%) **	195 (50.91%) **	63 (82.89%) **	0.000	0.203
Clinical pharmacy	239 (97.55%)	373 (97.39%)	73 (96.05%)	0.771	0.027
Pharmaceutical care	226 (92.24%)	366 (95.56%)	71 (93.42%)	0.214	0.066
Pathology and therapeutics	184 (75.10%) **	264 (68.93%) **	69 (90.79%) **	0.000	0.151
Computer and internet skills	160 (65.31%)	238 (62.14%)	48 (63.16%)	0.724	0.300
Interpersonal communication	155 (63.27%)	256 (66.84%)	59 (77.63%)	0.067	0.088
Traditional Chinese medicine	146 (59.59%) **	221 (57.70%) **	59 (77.63%) **	0.005	0.123
Pharmacoeconomics	140 (57.14%)	233 (60.84%)	51 (67.11%)	0.282	0.060
Pharmacoepidemiology	161 (65.71%) **	249 (65.01%) **	63 (82.89%) **	0.008	0.117
Evidence-based medicine	149 (60.82%) **	262 (68.41%) **	63 (82.89%) **	0.001	0.137
Molecular biology and genomics	130 (53.06%)	193 (50.39%) **	52 (68.42% )**	0.016	0.109

Note: * *p* < 0.05; ** *p* < 0.01 (chi-square test with post hoc multiple comparisons). The table presents the number and percentage of participants with reported training coverage in each knowledge and skill domain.

**Table 11:** Self-reported training coverage of knowledge and skill domains by Clinical Pharmacist Training Certificate and Clinical Pharmacist Instructor Certificate.

	Clinical Pharmacist Training Certificate		*p* Value	Cramer’s V	Clinical Pharmacist Instructor Certificate		*p* Value	Cramer’s V
	No	Yes			No	Yes		
Prescription review	222 (94.87%)	444 (94.47%)	0.823	0.008	593 (94.43%)	73 (96.05%)	0.554	0.022
Drug information retrieval and evaluation	199 (85.04%)	377 (80.21%)	0.118	0.059	511 (81.37%)	65 (85.53%)	0.375	0.033
Rational drug use	148 (63.25%)	262 (55.74%)	0.057	0.072	359 (57.17%)	51 (67.11%)	0.097	0.063
Clinical pharmacy	230 (98.29%)	455 (96.81%)	0.253	0.043	611 (97.29%)	74 (97.37%)	0.969	0.001
Pharmaceutical care	222 (94.87%)	441 (93.83%)	0.578	0.021	588 (93.63%)	75 (98.68%)	0.076	0.067
Pathology and therapeutics	182 (77.78%)	335 (71.28%)	0.066	0.069	457 (72.77%)	60 (78.95%)	0.250	0.043
Computer and internet skills	143 (61.11%)	303 (64.47%)	0.384	0.033	392 (62.42%)	54 (71.05%)	0.140	0.056
Interpersonal communication	142 (60.68%) *	328 (69.79%) *	0.016	0.091	407 (64.81%) **	63 (82.89%) **	0.002	0.119
Traditional Chinese medicine	148 (63.25%)	278 (59.15%)	0.295	0.040	376 (59.87%)	50 (65.79%)	0.319	0.037
Pharmacoeconomics	141 (60.26%)	283 (60.21%)	0.991	0.001	373 (59.39%)	51 (67.11%)	0.150	0.054
Pharmacoepidemiology	166 (70.94%)	307 (65.32%)	0.135	0.056	420 (66.88%)	53 (69.74%)	0.616	0.019
Evidence-based medicine	158 (67.52%)	316 (67.23%)	0.939	0.003	424 (67.52%)	50 (65.79%)	0.762	0.011
Molecular biology and genomics	129 (55.13%)	246 (52.34%)	0.485	0.026	328 (52.23%)	47 (61.84%)	0.113	0.060

Note: * *p* < 0.05; ** *p* < 0.01 (chi-square test). The table presents the number and percentage of participants with reported training coverage in each knowledge and skill domain.

**Table 12:** Self-reported training coverage of knowledge and skill domains by Clinical Pharmacist Advanced Training Certificate.

	Clinical Pharmacist Advanced Training Certificate		*p* Value	Cramer’s V
	No	Yes		
Prescription review	376 (94.00%)	290 (95.39%)	0.417	0.031
Drug information retrieval and evaluation	309 (77.25%) **	267 (87.83%) **	0.000	0.136
Rational drug use	201 (50.25%) **	209 (68.75%) **	0.000	0.186
Clinical pharmacy	389 (97.25%)	296 (97.37%)	0.923	0.004
Pharmaceutical care	378 (94.50%)	285 (93.75%)	0.674	0.016
Pathology and therapeutics	269 (67.25%) **	248 (81.58%) **	0.000	0.161
Computer and internet skills	250 (62.50%)	196 (64.47%)	0.590	0.020
Interpersonal communication	277 (69.25%)	193 (63.49%)	0.108	0.061
Traditional Chinese medicine	224 (56.00%) **	202 (66.45%) **	0.005	0.106
Pharmacoeconomics	235 (58.75%)	189 (62.17%)	0.358	0.035
Pharmacoepidemiology	247 (61.75%) **	226 (74.34%) **	0.000	0.133
Evidence-based medicine	265 (66.25%)	209 (68.75%)	0.484	0.026
Molecular biology and genomics	199 (49.75%) *	176 (57.89%) *	0.032	0.081

Note: * *p* < 0.05; ** *p* < 0.01 (chi-square test). The table presents the number and percentage of participants with reported training coverage in each knowledge and skill domain.

## Data Availability

The anonymized datasets generated and analyzed during the current study are available from the corresponding author upon reasonable request. The original questionnaire is also available as a Supplementary File. All data have been de-identified to ensure participant privacy and confidentiality.

## Supplementary Materials

The following supporting information can be downloaded at: https://www.mdpi.com/article/10.3390/healthcare14162622/s1, File S1: Questionnaire Items and Variable Coding Rules Used in This Study. File S2: Table S1. Questionnaire screening and data quality control procedures. Table S2. Comparison of characteristics between included participants and eligible excluded participants. Table S3. Bonferroni-adjusted post-hoc pairwise comparisons of training coverage across subgroups. Table S4. Self-reported training coverage of knowledge and skill domains by highest educational qualification.

## Abbreviations

The following abbreviations are used in this manuscript:

CE	Continuing Education
FIP	International Pharmaceutical Federation
GDP	gross domestic product
SMDs	standardized mean differences
SD	standard deviation
ACCP	American College of Clinical Pharmacy
ASHP	American Society of Health-System Pharmacists
PGx	pharmacogenomics
COVID-19	coronavirus disease 2019
