# Supplementary material for: Training Status and Self-Reported Training Coverage of Clinical Pharmacists Across Chinese Secondary and Tertiary Hospitals: A Cross-Sectional Survey
Source: Healthcare (Basel). 2026 Aug 19;14(16):2622. doi: 10.3390/healthcare14162622 (PMC13512099; doi:10.3390/healthcare14162622)
Supplement: Supplementary file 1 [file healthcare-14-02622-s001.zip › Supplementary File S1.pdf]

Questionnaire Items and Variable Coding Rules Used in This Study

This appendix describes the original questionnaire items used in the present study and the corresponding methods for variable construction.

The data for this study were derived from a nationwide comprehensive survey conducted in 2019 on the construction of the pharmaceutical care system and the implementation of pharmaceutical care in secondary and tertiary hospitals in China. The original survey consisted of eight sub-questionnaires, with respondents including survey investigators, hospital managers, heads of pharmacy departments, clinical pharmacists, physicians, other pharmacy professionals, and patients. The clinical pharmacist questionnaire included multiple modules, such as demographic information, training experience, work responsibilities, career development, barriers to pharmaceutical care, and occupational health status, involving a large number of survey items.

Since the present study focuses on the training status and self-reported training coverage across knowledge and skill domains among clinical pharmacists, this appendix includes only questionnaire items directly related to the objectives and analyses of the present study.

To help reviewers understand the source of variables and the data processing procedures, this appendix includes the following two parts:

Part I: Original Questionnaire Items Used in This Study

This section lists all original questionnaire items and response options included in the analyses of this study, including demographic characteristics, training experience, training qualifications, training certificates, and self-reported training coverage across knowledge and skill domains.

Part II: Variable Definitions and Coding Rules

This section provides detailed information on the source of variables, classification methods, variable recoding rules, and coding strategies used in the statistical analyses. For variables constructed from multiple original questionnaire items, the specific construction process is described to ensure the reproducibility and transparency of the study findings.

The contents presented in this appendix include only the questionnaire items and variables directly used in the present study. Other modules of the original comprehensive survey that were not related to the objectives of this manuscript are not included.

Part I: Original Questionnaire Items Used in This Study

Informed Consent

Dear Clinical Pharmacist,

Thank you for participating in this survey.

The purpose of this study is to investigate clinical pharmacists' training experiences, training status, and self-reported training coverage across knowledge and skill domains in secondary and tertiary hospitals in China. The findings of this study may provide evidence for improving clinical pharmacist training programs and continuing professional education.

This survey is conducted anonymously and does not collect personal information that could directly identify individual participants. All information provided will be used solely for academic research purposes and will not be used for any commercial purpose. Study results will be reported only in aggregate form, and individual responses will remain confidential.

Participation in this study is entirely voluntary. You may decline to participate or discontinue the questionnaire at any time without any negative consequences. This study does not involve clinical trials, medical interventions, or any activities that may pose risks to participants.

By completing and submitting this questionnaire, you acknowledge that you have read and understood the information above and voluntarily agree to participate in this study.

Thank you for your support and cooperation.

No.	Question	Response Options
1	What is the full name of your hospital?	___________
2	What is your gender?	☐ Male ☐ Female
3	What is your age?	_____ years
4	How many years have you worked in your current position or a similar position?	_____ years
5	What is your professional title?	☐ Junior ☐ Intermediate ☐ Associate Senior □ Senior
6	What is your highest educational qualification?	☐ Below Bachelor's Degree ☐ Bachelor's Degree ☐ Master's Degree ☐ Doctoral Degree
7	What qualifications do you hold for working as a clinical pharmacist? (Multiple Choice)	☐ National Specialized Clinical Pharmacist Training ☐ National General Clinical Pharmacist Training ☐ Provincial Specialized Clinical Pharmacist Training ☐ Provincial General Clinical Pharmacist Training ☐ No Formal Training ☐ No training of

No.	Question	Response Options
		any kind
8	What clinical pharmacist training programs or certificates have you obtained? (Multiple Choice)	□ Clinical Pharmacist Training Certificate □ Clinical Pharmacist Advanced Training Certificate □ Clinical Pharmacist Instructor Certificate
9	I have attended courses or training programs in the following knowledge or skill areas:	
(1)	Prescription review	□ Strongly Disagree □ Disagree □ Uncertain □ Agree □ Strongly Agree
(2)	Drug information retrieval and evaluation	□ Strongly Disagree □ Disagree □ Uncertain □ Agree □ Strongly Agree
(3)	Rational drug use	□ Strongly Disagree □ Disagree □ Uncertain □ Agree □ Strongly Agree
(4)	Clinical pharmacy	□ Strongly Disagree □ Disagree □ Uncertain □ Agree □ Strongly Agree
(5)	Pharmaceutical care	□ Strongly Disagree □ Disagree □ Uncertain □ Agree □ Strongly Agree
(6)	Pathology and therapeutics	□ Strongly Disagree □ Disagree □ Uncertain □ Agree □ Strongly Agree
(7)	Computer and internet skills	□ Strongly Disagree □ Disagree □ Uncertain □ Agree □ Strongly Agree
(8)	Interpersonal communication	□ Strongly Disagree □ Disagree □ Uncertain □ Agree □ Strongly Agree
(9)	Traditional Chinese Medicine (TCM)	□ Strongly Disagree □ Disagree □ Uncertain □ Agree □ Strongly Agree
(10)	Pharmacoeconomics	□ Strongly Disagree □ Disagree □ Uncertain □ Agree □ Strongly Agree
(11)	Pharmacoepidemiology	□ Strongly Disagree □ Disagree □ Uncertain □ Agree □ Strongly Agree
(12)	Evidence-Based Medicine (EBM)	□ Strongly Disagree □ Disagree □ Uncertain □ Agree □ Strongly Agree
(13)	Molecular Biology and Genomics	□ Strongly Disagree □ Disagree □ Uncertain □ Agree □ Strongly Agree

Part II: Variable Definitions and Coding Rules

Variable in Manuscript	Original Questionnaire Item	Variable Type	Coding Rule and Description
Hospital Level	Q1	Grouping Variable	Hospital level was determined based on the hospital name provided by the respondent through verification using the hospital's official website, the National Health Commission of China, and other publicly available sources. Hospitals were classified as secondary hospitals or tertiary hospitals.
Hospital Type	Q1	Grouping Variable	Hospital type was determined based on the hospital name provided by the respondent through verification using the hospital's official website and other publicly available sources. Hospitals were classified as general hospitals or specialty hospitals or Traditional Chinese medicine / integrated Chinese–Western medicine hospital.
Gender	Q2	Grouping Variable	Male = 0; Female = 1.
Age Group	Q3	Grouping Variable	18–29 years = 0; 30–39 years = 1; 40–49 years = 2; ≥50 years = 3.
Years of Practice	Q4	Grouping Variable	0-10 years = 0; 10–20 years = 1; ≥20 years = 2. Used for subgroup analysis.
Professional Title	Q5	Grouping Variable	Junior = 0; Intermediate = 1; Associate Senior = 2; Senior = 3.
Highest Educational Level	Q6	Grouping Variable	Below Bachelor's degree = 0; Bachelor's degree = 1; Master's degree = 2; Doctoral degree = 3.
Training Level	Q7	Grouping Variable	The original item was a multiple-choice question (National Specialized Training, National General Training, Provincial Specialized Training, Provincial General Training). Training level was classified into national-level training and provincial-level training. The national-level training group included pharmacists who had received only national-level training and those who had received both national-level and provincial-level training. The provincial-level training group included only pharmacists who had

**Variable in Manuscript**	**Original Questionnaire Item**	**Variable Type**	**Coding Rule and Description**
			received provincial-level training only.
Training Type	Q7	Grouping Variable	The original item was a multiple-choice question (National Specialized Training, National General Training, Provincial Specialized Training, Provincial General Training). Training type was classified into three categories: general training, specialized training, and both general and specialized training. The general training group included pharmacists who had received only general training; the specialized training group included pharmacists who had received only specialized training; and the combined group included pharmacists who had received both general and specialized training.
Clinical Pharmacist Training Certificate	Q8	Grouping Variable	Selected = Yes; Not selected = No.
Clinical Pharmacist Advanced Training Certificate	Q8	Grouping Variable	Selected = Yes; Not selected = No.
Clinical Pharmacist Instructor Certificate	Q8	Grouping Variable	Selected = Yes; Not selected = No.

Note: Certificate categories were defined according to certificate types within the national clinical pharmacist training system. Because administrative authorities changed over time, certificates issued during different periods were classified based on their certificate category rather than the issuing institution.
