# Supplementary material for: Training Status and Self-Reported Training Coverage of Clinical Pharmacists Across Chinese Secondary and Tertiary Hospitals: A Cross-Sectional Survey
Source: Healthcare (Basel). 2026 Aug 19;14(16):2622. doi: 10.3390/healthcare14162622 (PMC13512099; doi:10.3390/healthcare14162622)
Supplement: Supplementary file 1 [file healthcare-14-02622-s001.zip › Supplementary File S2.pdf]

Supplementary Table S1. Questionnaire screening and data quality control procedures

Data processing step	Screening criteria	Excluded (n)	Remaining (n)
Returned questionnaires	Completed and submitted questionnaires	—	1300
Eligibility assessment	Participants not meeting inclusion criteria (not full-time clinical pharmacists working in **secondary** or **tertiary** hospitals)	92	1208
Duplicate record verification	Duplicate records identified during database management	1	1207
Response validity assessment	Multiple responses to single-choice questions	77	1130
	Out-of-range responses for key variables	29	1101
Skip instruction compliance assessment	Failure to follow predefined skip instructions	74	1027
Logical consistency assessment	Mutually incompatible responses between related items	82	945
	Implausible demographic information (e.g., inconsistent age and years of professional experience)	26	919
	Contradictory training-related responses (e.g., selecting "no training of any kind" while subsequently reporting participation in one or more knowledge and skill training domains)	215	704
Final analytic sample	Questionnaires retained after quality control		704

Supplementary Table S2. Comparison of characteristics between included participants and eligible excluded participants

Variable	Included (n=704)	Excluded (n=503)	P value	SMD
**Age （Mean ± SD）**	35.69±6.62	35.33±7.81	0.085	0.051
**Years of professional experience （Mean ± SD）**	9.52±6.23	10.39±8.23	0.974	0.121
**Hospital level**			0.000	0.273
Secondary	375	200		
Tertiary	329	303		
**Hospital type**			0.021	
General hospital	587	389		0.153
Specialty hospital	40	33		0.037
Traditional Chinese medicine / integrated Chinese–Western medicine hospital	77	81		0.152
**Gender**			0.442	0.045
Male	253	170		
Female	451	333		
**Professional title**			0.000	
Junior	208	220		0.298
Intermediate	403	215		0.293
Associate senior	81	56		0.012
Senior	12	12		0.048
**Highest educational qualification**			0.013	
Below bachelor's degree	38	51		0.178
Bachelor's degree	448	317		0.013
Master's degree	213	131		0.094
Doctoral degree	5	4		0.01

Supplementary Table S3. Bonferroni-adjusted post-hoc pairwise comparisons of training coverage across subgroups

Training domain	Subgroup variable	Comparison	Raw P value	Bonferroni-adjusted P value
Drug information retrieval and evaluation	Training type	General training only vs Specialist training only	0.034	0.102
		General training only vs Both general and specialist training	0.192	0.576
		Specialist training only vs Both general and specialist training	0.011	0.033
Rational drug use	Age	18-29 vs 30-39	0.053	0.318
		18-29 vs 40-49	0.805	1
		18-29 vs 50+	0.065	0.39
		30-39 vs 40-49	0.002	0.012
		30-39 vs 50+	0.001	0.006
		40-49 vs 50+	0.069	0.414
	Years of work experience	0-10 vs 10-20	0.079	0.237
		0-10 vs 20+	0.001	0.003
		10-20 vs 20+	0.045	0.135
	Training type	General training only vs Specialist training only	0.006	0.018
		General training only vs Both general and specialist training	0.001	0.003
		Specialist training only vs Both general and specialist training	0.001	0.003
Pharmaceutical care	Age	18-29 vs 30-39	0.475	1
		18-29 vs 40-49	0.315	1
		18-29 vs 50+	0.970	1
		30-39 vs 40-49	0.004	0.024
		30-39 vs 50+	0.567	1
		40-49 vs 50+	0.479	1
Pathology and therapeutics	Training type	General training only vs Specialist training only	0.095	0.285
		General training only vs Both general and specialist training	0.003	0.009
		Specialist training only vs Both general and specialist training	0.001	0.003
Traditional Chinese medicine	Hospital type	General hospitals vs Specialist hospitals	0.218	0.654
		General hospitals vs Traditional Chinese medicine / integrated Chinese–Western medicine hospitals	0.001	0.003

Training domain	Subgroup variable	Comparison	Raw P value	Bonferroni-adjusted P value
		Specialist hospitals vs Traditional Chinese medicine / integrated Chinese–Western medicine hospitals	0.164	0.492
	Training type	General training only vs Specialist training only	0.639	1
		General training only vs Both general and specialist training	0.004	0.012
		Specialist training only vs Both general and specialist training	0.001	0.003
Pharmacoepidemiology	Professional title	Junior vs Intermediate	0.006	0.036
		Junior vs Associate senior	0.901	1
		Junior vs Senior	0.912	1
		Intermediate vs Associate senior	0.077	0.462
		Intermediate vs Senior	0.378	1
		Associate senior vs Senior	0.875	1
	Training type	General training only vs Specialist training only	0.857	1
		General training only vs Both general and specialist training	0.004	0.012
		Specialist training only vs Both general and specialist training	0.002	0.006
Evidence-based medicine	Training type	General training only vs Specialist training only	0.051	0.153
		General training only vs Both general and specialist training	0.001	0.003
		Specialist training only vs Both general and specialist training	0.011	0.033
Molecular biology and genomics	Age	18-29 vs 30-39	0.004	0.024
		18-29 vs 40-49	0.149	0.894
		18-29 vs 50+	0.104	0.624
		30-39 vs 40-49	0.081	0.486
		30-39 vs 50+	0.844	1
		40-49 vs 50+	0.505	1
	Training type	General training only vs Specialist training only	0.514	1
		General training only vs Both general and specialist training	0.018	0.054
		Specialist training only vs Both general and specialist training	0.004	0.012

Supplementary Table S4. Self-reported training coverage of knowledge and skill domains by highest educational qualification

	Highest educational qualification					
	Below bachelor's degree	Bachelor's degree	Master's degree	Doctoral degree	**P value**	**Cramer's V**
**Prescription review**	34(89.47%)	420(93.75%)	207(97.18%)	5(100%)	0.129	0.090
**Drug information retrieval and evaluation**	32(84.21%)	365(81.47%)	174(81.69%)	5(100%)	0.730	0.043
**Rational drug use**	26(68.42%)	260(58.04%)	121(56.81%)	3(60.00%)	0.612	0.051
**Clinical pharmacy**	37(97.37%)	437(97.54%)	206(96.71%)	5(100%)	0.914	0.027
**Pharmaceutical care**	35(92.11%)	420(93.75%)	203(95.31%)	5(100.00%)	0.741	0.042
**Pathology and therapeutics**	26(68.42%)	337(75.22%)	150(70.42%)	4(80.00%)	0.508	0.058
**Computer and internet skills**	24(63.16%)	281(62.72%)	137(64.32%)	4(80.00%)	0.859	0.033
**Interpersonal communication**	23(60.53%)	296(66.07%)	148(69.48%)	3(60.00%)	0.665	0.047
**Traditional Chinese medicine**	27(71.05%)	273(60.94%)	122(57.28%)	4(80.00%)	0.317	0.071
**Pharmacoeconomics**	19(50.00%)	265(59.15%)	136(63.85%)	4(80.00%)	0.277	0.074
**Pharmacoepidemiology**	23(60.53%)	306(68.30%)	142(66.67%)	2(40.00%)	0.437	0.062
**Evidence-based medicine**	26(68.42%)	295(65.85%)	149(69.95%)	4(80.00%)	0.683	0.046
**Molecular biology and genomics**	17(44.74%)	231(51.56%)	123(57.75%)	4(80.00%)	0.188	0.083

Note: The table presents only the number and percentage of participants with covered training.
